# Insufficient Sleep Syndrome in Childhood

**DOI:** 10.3390/children12010019

**Published:** 2024-12-26

**Authors:** Teruhisa Miike

**Affiliations:** School of Medicine, Kumamoto University, Kumamoto 860-8556, Japan; t.miike4297@gmail.com

**Keywords:** insufficient sleep syndrome, infants and early childhood, nighttime basic sleep duration, circadian rhythm, sleep hygiene, melatonin

## Abstract

Sleep disorders in children have a negative impact on mental and physical development, and a lack of sleep is one of the most important problems in infancy. At the age when naps are commonly accepted, the judgment of whether the amount of sleep is adequate has been based on the total amount of sleep per day. In other words, the idea is that even if the amount of sleep at night is insuﬃcient, it is not considered insuﬃcient if it is compensated for by taking a long nap or sleeping late on weekend mornings. However, these lifestyle habits disrupt the circadian rhythm and cause social jet lag, which is not appropriate for healthy mental and physical development. Therefore, in this review, I present the average required nighCime basic sleep duration (NBSD) of 10 h for Japanese and 11 h for Caucasian children as a judgment standard. (1) If the child sleeps less than 8 h at night, and (2) if the child sleeps less than 9 h at night or 30 to 60 min less than the required NBSD, immediate treatment is recommended. I also discuss briefly how to address sleep insufficiency in childhood.

## 1. Introduction

Sleep is necessary to maintain good brain performance during wakefulness [1]. The biological functions of sleep can be divided into four categories: (1) metabolism and functional recovery, (2) defense and response to injury, (3) neurodynamics and neuroplasticity, and (4) biological periodicity and the timing of biological processes [2].

From a pediatrician’s point of view, sleep is a time for (1) brain creation during fetal development, (2) brain development after birth, (3) the protection of brain function through maintenance, (4) the accumulation of knowledge, and (5) the further enhancement of cognitive function [3]. Such information indicates the importance of sleep for children, and there is concern that even slight sleep deprivation during infancy may accumulate and have a negative impact on children’s mental and physical development [4].

The theme of this Special Issue, namely, “insufficient sleep syndrome (ISS)”, is a symptom commonly seen in children living in modern society due to chronic sleep deprivation (CSD) and may cause problems not only with sleep itself, but also with various functions of the entire body. Humans have survived by learning the rhythm of going to sleep when it gets dark and waking up when the Sun rises. This principle is the same in modern society, where people are expected to live according to the daily schedules of school and society from morning to evening.

In modern society, even though the time we should wake up has not changed in the past several decades, people have started going to bed later and a nocturnal lifestyle has taken hold. This means that people’s nighttime sleep duration is gradually being reduced. The shift to a late-night lifestyle is also affecting children, with a sharp increase in the reported number of children suffering from nocturnal sleep deprivation [3].

In particular, children’s activity times are closely related to childcare, kindergarten, and school life, and even now, school life requires them to wake up by around 7:00 a.m., regardless of country (Table 1).

Therefore, a living environment has been created in which many modern people, regardless of generation, are forced to suffer from chronic sleep deprivation. As mentioned above, sleep plays an important role in maintaining human life, and a lack of sleep clearly does not lead to people’s happiness. Here, I emphasize the importance of sleep deprivation, and, from a broader perspective, the circadian rhythm biological clock becomes a significant problem. The life rhythm associated with human sleep and wakefulness is controlled by the biological circadian clock mechanism and is not just related to sleep. It has major impacts on the autonomic nervous function [5,6,7], thermoregulation [8,9,10,11], hormone secretion [8,12], glucose/lipid metabolism/energy production [13,14,15], intestinal flora [16], and immune support function [17,18,19], and it is becoming necessary to consider it as an issue that affects all life-sustaining functions. These circadian rhythms, especially the sleep–wake rhythm, are established early after birth [12,20,21,22,23,24] and are known to have lifelong effects. The formation of inappropriate circadian rhythms leads to mental and physical health disorders, such as autonomic symptoms, glucose metabolism disorders, hyperthermia, adult metabolic diseases, heart disease, cancer, and dementia with age [25,26]. Recognizing that a lack of sleep, especially chronic sleep deprivation, is the first step in these various health problems and working to improve it will contribute to the healthy mental and physical development of children throughout their lives.

This is a cross-tabulation table comparing the time of falling asleep and the time of waking up in the morning between weekdays and weekends of subjects divided into several age groups. The average times, first quartiles, medians, and third quartiles are indicated by hours and minutes, and the SDs (standard deviations) are in minutes.

## 2. Childhood Sleep Insufficiency

The oldest record of sleep insufficiency in children may date back to 1937 [27]. Sleep deprivation causes various brain dysfunctions in both adults and children, and is associated with behavioral, cognitive, and physical problems and atypical early development [28]. However, even with reference to the International Classification of Sleep Disorders, Third Edition (ICSD-3d), the diagnostic criteria are clinical and it is difficult to establish clear diagnostic criteria. The problems can be summarized as follows: (1) Unbearable sleepiness occurs almost every day, and in children, behavioral abnormalities due to sleepiness are observed. (2) Because they are woken up by an alarm clock or family members in the morning, they are unable to get enough sleep at night, and often sleep longer on weekends. (3) The symptoms disappear when they obtain enough sleep. These are considered to be the main clinical symptoms required for diagnosis. The current situation is that the diagnosis is made based on the presence or absence of symptoms that are thought to be caused by sleep deprivation (daytime sleepiness, decreased concentration, decreased energy, general fatigue, etc.) and the nighttime sleep situation. Sleep disorders, including chronic sleep deprivation, are thought to cause declines in neural function; memory and learning; gene expression; neurogenesis; and cognitive, behavioral, and health functions through a variety of mechanisms. If these changes continue for a long period of time, excessive cellular stress may result, leading to widespread neuronal loss [29]. As mentioned above, sleep deprivation has been reported to cause various problems related to human life-sustaining functions [30,31,32].

### 2.1. Nighttime Basic Sleep Duration (NBSD)

To assess whether or not a child is sleep deficient, it is necessary to determine the appropriate duration of sleep each child needs. There are several excellent reports on the sleep characteristics of infants and toddlers, which consistently reported the following for nighttime sleep duration: in Switzerland [33], the average nighttime sleep duration for children aged 1–5 years is more than 11 h (11.7–11.1 h); in the UK [34], the average nighttime sleep duration for children aged 1–6 years is approximately 11 h; and in Canada [20], the average nighttime sleep duration for children aged 6 months–4 years is 10 h (47.6%) or 11 h (42.8%), respectively. Interestingly, these papers report that while the total sleep duration decreases with age, the nighttime sleep duration remains almost unchanged. A Swiss report [33] found that the nighttime sleep duration increases slightly from infancy to 1–3 years of age, and then returns to infant levels by 5–6 years of age. This can be interpreted as no significant increase or decrease in the 1–6-year-old group.

Although none of these reports particularly emphasize this nearly constant nighttime sleep duration, the data from these reports commonly show that the nighttime sleep duration is relatively stable from infancy to age 6 years. On the other hand, the daytime sleep duration gradually decreases and disappears by 2–5 years [35], and at the latest by age 7 years [36].

**Figure 1:**
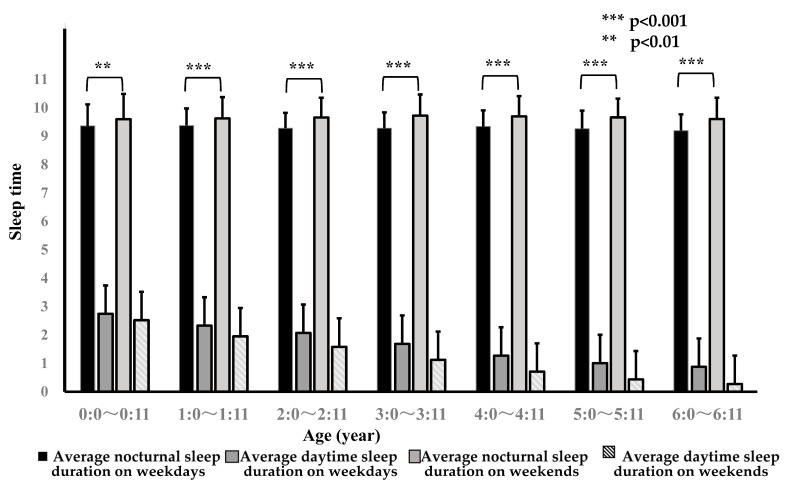
Nocturnal sleep duration and daytime sleep duration of various age groups on weekdays and weekends [3].

Recently, I reported that Japanese infants and toddlers need an average of about 10 h of nighttime sleep regardless of age (Figure 1). As children grow, the total daily sleep duration decreases because the daytime sleep duration decreases, but the nighttime sleep duration remains almost constant at about 10 h from ages 1 to 6 years. Therefore, I considered this average of 10 h to be the basic sleep duration required for children’s daily rhythm and decided to call it the nighttime basic sleep duration (NBSD) [3].

In conclusion, the required nighttime sleep duration (NBSD) for children aged 1 to 6 years is an average of 10 h for Japanese children and 10–11 h for Caucasian children, with most children needing 11 h.

The bar graph shows the average durations, and the lines extending above the bar graphs show the SDs (standard deviations). The four bars of each age group represent, from left to right, the nocturnal sleep duration on weekdays, daytime sleep duration on weekdays, nocturnal sleep duration on weekends, and daytime sleep duration on weekends.

### 2.2. Insufficient Sleep Means Lack of Nocturnal Sleep

For adults, nighttime sleep deprivation is generally considered to mean insufficient sleep. The appropriate sleep duration for age groups that no longer take naps is often cited as proposed by the American Academy of Sleep Medicine [37] and the American Sleep Foundation [38] (Table 2), but, of course, nap time is not included in this age group. However, in reports on children under 6 who take naps, the total sleep time per day is somehow presented as an indicator of the adequacy or insufficiency of the sleep duration (Table 2).

I have not seen any papers that raise this issue, but I wonder whether this is an appropriate indicator for children. I would like to emphasize the importance of considering nighttime sleep deprivation as sleep deprivation, rather than the total sleep time per day, for the following reasons:

(1) There is a certain amount of nighttime sleep that children actually need [3,20,33,34].
(2) Even a small amount of sleep deprivation accumulates and causes problems [4].
(3) Nighttime sleep deprivation needs to be compensated for somewhere during the day, resulting in irregular and long naps and long sleep on weekend mornings [3].
(4) The need for naps continues even when children enter elementary school, as the accumulation of sleep deprivation and the shift in daily rhythms leads to the disruption of daily life and may cause problems in future school and social life. A lifestyle in which sleep deprivation during the weekdays is compensated for by sleeping late into the morning on weekends may lead to “social jet lag” [3,39,40].
(5) If sleep deprivation is insufficiently compensated, the accumulation of sleep deprivation may lead to sleep debt, which may lead to various dysfunctions of the entire body, including decreased brain function [29,30,31,32].

In conclusion, using the total daily sleep time as an indicator to evaluate the adequacy or insufficiency of sleep time means accepting a lifestyle in which a lack of nighttime sleep is compensated for by naps and morning sleep-ins on weekends (and holidays), which, as mentioned above, does not lead to the formation of an appropriate biological clock to maintain children’s mental and physical health. Recently, it has been reported that nighttime sleep, as part of overall sleep, has a significant impact on children’s mental and physical development [3,41,42,43,44]. Recent reports on the importance of nighttime sleep for children seem to be on target. Therefore, even for children under 6 years of age, attention should be paid to whether nighttime sleep is sufficient, and it is not recommended that sleep be evaluated based on the total daily sleep time, including naps. Paruthi et al. [37] emphasized the importance of “regular sleep” and stated that it is associated with better health outcomes, such as improved attention, behavior, learning, memory, emotional regulation, quality of life, and mental and physical health. I would like to emphasize that establishing consistent daily routines (sufficient nighttime sleep and appropriate nap time and length) is an important factor for children’s physical and mental development [3].

## 3. Diagnostic Criteria for ISS in Children Aged 1–6 (Proposal)

Until now, the appropriate amount of sleep for infants who require naps has been assessed based on the total daily sleep time, including naps [45,46]. However, as mentioned above, sleep deprivation in infants and toddlers should be considered based on the nighttime sleep duration. When assessing sleep for this age group, first, it is important to recognize the amount of nighttime sleep required (NBSD) for each child. Each child’s NBSD can be calculated by recording the child’s daytime rhythm using actigraphy or a recommended 2-week sleep chart, if possible. If these methods are not available, a rough assessment can be made by waiting until the child wakes up on their own on a weekend morning. Since it is not practical to use actigraphy in every home and there are situations in which actigraphy does not always provide accurate sleep duration data [3], clinically, recording a sleep chart is sufficient. As for assessment, a child’s sleep duration of less than 8 h at night appears to clearly signify sleep deprivation [47,48]. What is noteworthy about the sleep of infants and young children is undoubtedly its relationship with their mental and physical development. There are many papers on sleep disorders in children with autism spectrum disorders, but none of them specify the length of sleep that should be of concern, and most simply state that the sleep duration is “short”. Petit D et al. [47] reported the following interesting and important information: (1) Children who slept less than 8 h per night at 2.5 years of age and later normalized were three to five times more likely to perform below the class average in reading, writing, mathematics, and science than children who slept adequately (10 to 11 h per night). (2) Children who slept about 9 h per night throughout childhood were two to three times more likely to perform below the class average in mathematics and science. What is also interesting is that the amount of sleep at age 10 was not correlated with academic achievement.

These results indicate that adequate sleep during early childhood is required to fine-tune the functioning necessary for later academic achievement. Recently, we reported that less than 8 h of nighttime sleep, especially in children under 3 years of age, may indicate the possibility of developing ASD in the future [48]. Although some reports have specifically pointed out the risk of less than 7 h of nighttime sleep, the age range of the study was 4 to 18 years, and it was considered not very appropriate to evaluate the sleep time of infants and young children [49]. On the other hand, although the age distribution is unknown, it has been reported that the total daily sleep time of children with ASD is 32.8 min shorter than that of typically developing children [45]. Another study [50] reported that the sleep time of 52 children with ASD (3–10 years old) who were highly irritable was 60 min shorter than that of children with low irritability, and children with high stereotypy slept 75 min less than those with low stereotypy. In other words, the shorter the sleep time, the more irritable and stereotypic the child was. Based on this information, I would like to propose the following criteria for determining whether infants and toddlers (1–6 years old) are sleep-deprived:

(1) If the child sleeps less than 8 h at night, there is a high possibility that the child is sleep-deprived, which may have a negative impact on the child’s physical and mental development, and immediate treatment is required, including medication.
(2) If the child sleeps less than 9 h at night or 30 to 60 min less than the required NBSD, chronic sleep deprivation is suspected and sleep debt is a concern. In addition, if the child has the following clinical symptoms, immediate treatment is recommended: (1) unbearable sleepiness almost every day, (2) behavioral abnormalities due to sleepiness are observed, (3) the child is woken up in the morning by an alarm clock or family members, (4) the child tends to sleep longer on weekends, and (5) the symptoms disappear when the child acquires enough sleep.

## 4. Sleep Information for Early Childhood Is Important

Although sleep disorders in infancy and early childhood often improve gradually, it is thought that sleep disorders in infancy and early childhood themselves may have a significant impact on subsequent physical and mental development [3,47,48].

More importantly, it has been reported that the formation of an appropriate biological clock from the fetal to infant stages has an impact on maintaining mental and physical health throughout one’s life [25,26]. In simple terms, it has been reported that the daily life of a mother during pregnancy affects the programming of the fetus’s biological clock [22], as well as its mental and physical functions, and that the formation of an inappropriate biological clock has various adverse effects on mental and physical functions as the child grows after birth [25,26].

Since the circadian rhythm that is established after birth is formed based on ultradian rhythms [51], it can be interpreted that the formation of appropriate ultradian rhythms during fetal development leads to the formation of appropriate circadian rhythms after birth, which is the basis for maintaining healthy mental and physical functions throughout one’s life.

Conversely, if circadian rhythms are not properly formed, they can cause various mental and physical health problems in the future. For example, disruptions to the biological clock, including sleep disorders, are thought to lead to problems such as neurodevelopmental disorders, school absenteeism, and depression in the early stages; after this, adult metabolic diseases, such as kidney and heart diseases, type II diabetes, other mental disorders, and even cancer and dementia, develop with age [25,26]. Therefore, if sleep disorders are observed in infancy, it is not appropriate to think that it is sufficient to simply continue to monitor the condition if the sleep disorder improves as the child grows. If a sleep disorder is identified in infancy, it is recommended that the child receive appropriate treatment as soon as possible.

## 5. Sleep Deprivation Habits Starting in Infancy

The displacement of bedtime and wake times for later hours on free days starts at an early age [52]. A survey of nursery school children conducted from 2012 to 2014 revealed that children’s bedtimes were becoming later, on average, from around 9:00 p.m. to around 9:30 p.m. from immediately after birth until around the age of two [3]. As mentioned above, children need about 10 to 11 h of sleep at night (NBSD), and in modern society, actual wake-up times are generally before 7:00 a.m. By simple calculation, children who fall asleep after 9:30 p.m. may be lacking in sleep by more than 30 min a day.

**Figure 2:**
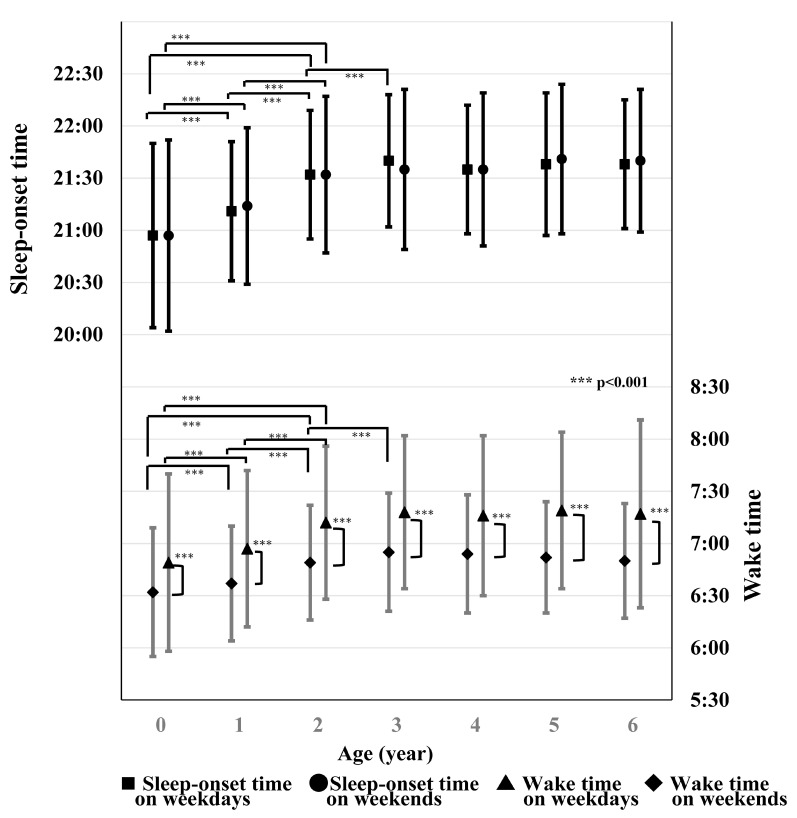
Night bedtime and morning wake time for each age group on weekdays and at weekends [3].

Thus, a daily lack of sleep is a global problem, and since the authors’ data show that there is a difference of about 30 min between waking up on weekdays and waking up on weekends, it seems correct to think that children are gradually becoming more sleep-deprived each day [3] (Figure 2).

The point at the center of each vertical line represents the average time and the lines extending above and below the point represent the SD (standard deviation). The pairs of lines on the lower side represent the morning wake time of each age group, where the left line of each pair represents the wake time on weekdays and the right line of each pair represents the wake time on weekends. The pairs of lines on the upper side represent each age group’s bedtime, while the left line represents the bedtime on weekdays and the right line represents the sleep-onset time on weekends. The time scale for the bedtime is given on the left of the ordinate and that for the wake time is given on the right. Although the bedtimes became later with age by 2 years old (*p* < 0.001), those of the children older than 2 years did not change with age. No difference was found (*p* = 0.751) for the bedtimes of the same group between weekdays and weekends. On the other hand, the wake times of all the groups on weekends were significantly later (*p* < 0.001) than those on weekdays, and the difference increased with age.

Sleep deprivation among Japanese children occurs on a daily basis from birth, and this may accumulate; it is thought that they compensate for this by taking long naps on weekdays and longer sleep on weekend mornings, preventing the accumulation of sleep deprivation. Similar lifestyle habits have been confirmed almost all over the world, and have emerged as a common problem regardless of region or race [53,54,55,56,57,58]. Compared with several decades ago, people’s sleep start times have become significantly later in recent years, and it has been reported that more people are staying up late, especially since the COVID-19 pandemic [59,60,61]. However, it has also been reported that morning wake-up times have not changed in the past few decades. Naturally, this situation is expected to spread to children, and drastic measures are needed to address the lifestyle habits of modern children, whose sleep start times are delayed without changes in wake-up times.

From this information, it can be inferred that the lifestyle habits that lead to children’s lack of sleep are due to a mismatch between their bedtime and wake-up time, and that they are not obtaining the appropriate amount of nighttime sleep (NBSD: average 10–11 h) needed to wake up in the morning. This mismatch in lifestyle rhythms causes “social jet lag”, which interferes with children’s school and social lives.

## 6. Insufficient Sleep Syndrome Develops Due to Circadian Rhythm Disruption

Chronic sleep deprivation is the first step in circadian rhythm disruption. Sleep deprivation is defined as nocturnal sleep that is shorter than the recommended sleep duration for health (NBSD), and circadian misalignment is defined as being awake and active (including eating) when the biological clock demands sleep. Therefore, although there have been many reports in recent years stating that nocturnal lifestyles and sleep deprivation are associated with obesity, it is important to understand that this issue is not just focused on obesity, but is important in relation to other life-sustaining functions of the body [5,6,7,8,9,10,11,12,13,14,15,16,17,18,19].

As a result, the biological clock becomes misaligned and the circadian rhythm becomes disrupted. This is called chronodisruption, and the changes over time are as follows: everyday lack of sleep → sleep replenishment by taking long naps on weekdays and/or long sleep on weekend mornings → social jet lag (insufficient replenishment) → sleep debt → circadian rhythm sleep disorders, primarily DSWPD: onset of chronodisruption → withdrawal from school/social life.

## 7. Background of Children’s Nocturnal Sleep Deprivation

As mentioned above, the main background of children’s nocturnal sleep deprivation is thought to be (1) the late-night lifestyle that is widespread in modern society, making it difficult to ensure sufficient sleep time before waking up (often around 7:00 a.m.). In modern society, it cannot be denied that it is becoming more difficult for children to maintain a sleep-onset time of 8:00 p.m.–9:00 p.m., which is necessary to ensure the appropriate nighttime sleep time (NBSD: 10–11 h) required for their physical and mental growth and development by the time they wake up in the morning. (2) Alternatively, various reasons for difficulty in maintaining continuous nighttime sleep (long nighttime awakenings) can also be cited as background factors that cause sleep deprivation. These background factors include (1) daily habits, such as media use [62,63,64,65,66,67,68]; (2) difficulty falling asleep due to predisposing factors, lifestyle habits, and social background [69,70,71,72,73]; (3) frequent and/or long nighttime awakenings (sleep persistence disorder) [74,75]; and (4) other problems that can occur when a child’s NBSD is longer than 11 h. The longer the NBSD, the more likely the child will need to go to bed earlier, making it difficult to realistically ensure an appropriate time for falling asleep. Late sleep onset and long awakenings during nighttime sleep lead to sleep deprivation, which is compensated for by long/irregular sleep during the day and long sleep on weekend mornings, preventing the accumulation of sleep loss and maintenance of an appropriate daily rhythm [3].

## 8. Treatment for Insufficient Sleep Syndrome

I will leave the treatment to other papers, but I will provide a brief overview here. It is recommended that sleep disorders, especially those in infancy and early childhood, be addressed promptly and without delay [29,76,77,78]. It is known that addressing and treating sleep disorders in infancy not only promotes the child’s physical and mental development, but also contributes to maintaining the physical and mental health of the parents [79,80,81], so it is best not to leave the problem unattended even for a short period of time. For this reason, immediate solutions, including pharmacotherapy, are desirable [76,82]. It can be said that the solution lies in adjusting the sleep–wake circadian rhythm and correcting nighttime awakenings.

### 8.1. Sleep Hygiene

#### 8.1.1. Sleep Education

There are important reports of sleep education for mothers after giving birth [77,79], but sleep education for pregnant women is also needed [25,26,83]. I started the latter initiative in Sanjo city-Niigata in Japan in 2016, and although there was an improvement in that area, it has not spread nationwide as much as expected. As children grow older and are able to learn about sleep using picture books, they will begin to make efforts to establish a regular routine on their own. When they grow even older and enter elementary school, they can receive sleep education in class, and it has been proven that this can significantly reduce school absenteeism due to sleep disorders [84].

#### 8.1.2. Behavioral Sleep Intervention

Behavioral sleep interventions for infants and early childhood, such as settling and bedtime routines, are widely used training methods to improve sleep quality in children with sleep problems such as frequent nighttime awakenings, short sleep duration, and sleep-onset latency [82]. The settling methods are carried out in various ways, such as cry it out and fading [85,86,87]. The common effect of these methods is that the child calms down on his or her own, so-called “self-soothing” [21,88,89,90,91]. What matters is whether the method allows the infant to sleep continuously throughout the night with the right rhythm. Without a proper sleep rhythm, the child cannot develop a rhythm that adequately promotes attachment and emotional brain development. In other words, without a proper biological clock, attachment and balanced emotions cannot develop. Therefore, the evaluation of behavioral therapy should be based on whether the child is getting enough uninterrupted sleep and developing appropriate sleep onset and wake-up times [3,92].

### 8.2. Pharmacotherapy

Pediatricians generally tend to avoid pharmacotherapy in this age group, and many are opposed to it, but when comparing the adverse effects of sleep disorders on a child’s lifelong mental and physical development and health maintenance with the side effects of medication, I believe that this treatment option should clearly be emphasized. Needless to say, if pharmacotherapy is necessary, it should be selected carefully, and the minimum effective dose and appropriate administration period should be considered [76,82]. It has been reported that more than 50% of pediatricians use pharmacotherapy, including drugs that are used in adults but not recommended for children [93]. If sleep hygiene advice is ineffective, I recommend starting pharmacotherapy without hesitation. Kelsay [94] reported a clear schema of the pharmacological treatment of sleep disorders in children with allergic dermatitis. Melatonin is recommended as the first medication for delayed sleep onset, followed by antihistamines or clonidine depending on the efficacy to date. I have been sustaining a similar treatment since the early 1990s and can agree that it is an appropriate treatment. Various reports have been published about melatonin since its clinical use began [95,96,97], and although there have been reports of minor side effects [98,99], there are few concerns about its use in infants and young children when used appropriately. However, melatonin does not provide clinical benefits in infants at the usual dose (1.5 mg), so short-term use is recommended as early as possible between 12 and 24 months of age.

## 9. Conclusions

It is recommended that sleep disorders in childhood be addressed immediately, regardless of their age and condition. A lack of sleep is particularly detrimental, as it can lead to various mental and physical health problems in the future. The standard for judging whether a child is acquiring enough sleep should not be the total amount of sleep per day, but rather the presence or absence of a lack of nighttime sleep, just like in adults. As a standard for this judgment, I propose that the average nighttime basic sleep duration for Japanese infants and young children should be 10 h, and for Caucasian children, 11 h. Guardians should first determine their child’s exact NBSD and establish an appropriate bedtime so that this NBSD can be secured by the required morning wake-up time (around 7:00 a.m.), preventing the accumulation of insufficient sleep and deviations in daily rhythms, including social jet lag. This will help promote balanced mental and physical development in children and help them form an appropriate circadian rhythm biological clock to maintain lifelong mental and physical health.

## Figures and Tables

**Table 1:** Night bedtimes and morning wake times of various age groups on weekdays and at the weekend [3].

	Age	Number of Subjects	Sleep-Onset Time (hr:min)					Wake Time (hr:min)				
			Ave.	SD	1st Quartile	Median	3rd Quartile	Ave.	SD	1st Quartile	Median	3rd Quartile
Weekdays	0 mo. to 11 mos.	236	20:57	53	20:30	21:00	21:30	6:32	37	6:00	6:30	7:00
	1 yr. to 1 yr. and 11 mos.	1532	21:11	40	20:45	21:00	21:30	6:37	33	6:15	6:30	7:00
	2 yrs. to 2 yrs. & 11 mos.	1319	21:32	37	21:00	21:30	22:00	6:49	33	6:30	7:00	7:00
	3 yrs. to 3 yrs. & 11 mos.	842	21:40	38	21:15	21:30	22:00	6:55	34	6:30	7:00	7:15
	4 yrs. to 4 yrs. & 11 mos.	478	21:35	37	21:00	21:30	22:00	6:54	34	6:30	7:00	7:15
	5 yrs. to 5 yrs. & 11 mos.	355	21:38	41	21:00	21:30	22:00	6:52	32	6:30	7:00	7:00
	6 yrs. to 6 yrs. & 11 mos.	119	21:38	37	21:30	21:30	22:00	6:50	33	6:30	7:00	7:00
	All the age groups	4881	21:26	41	21:00	21:30	22:00	6:46	34	6:30	6:45	7:00
Weekends	0 mo. to 11 mos.	236	20:57	55	20:30	21:00	21:30	6:49	51	6:15	6:45	7:15
	1 yr. to 1 yr. & 11 mos.	1532	21:14	45	20:45	21:15	21:45	6:57	45	6:30	7:00	7:30
	2 yrs. to 2 yrs. & 11 mos.	1319	21:32	45	21:00	21:30	22:00	7:12	44	6:45	7:00	7:45
	3 yrs. to 3 yrs. & 11 mos.	842	21:35	46	21:00	21:30	22:00	7:18	44	6:49	7:15	7:45
	4 yrs. to 4 yrs. & 11 mos.	478	21:35	44	21:00	21:30	22:00	7:16	46	6:45	7:15	7:45
	5 yrs. to 5 yrs. & 11 mos.	355	21:41	43	21:15	21:45	22:00	7:19	45	7:00	7:15	7:45
	6 yrs. to 6 yrs. & 11 mos.	119	21:40	41	21:15	21:30	22:00	7:17	54	6:45	7:15	7:45
	All the age groups	4881	21:26	47	21:00	21:30	22:00	7:08	46	6:30	7:00	7:30

SD: standard deviation.

**Table 2:** Sleep duration recommendations (in hours) for children.

	National Sleep Foundation’s Sleep Time Duration Recommendations [38]			A Consensus Statement from the American Academy of Sleep Medicine [37]	
Age	May Be Appropriate	Recommended (h)	May Be Appropriate	Age	Recommended (h)
Newborns 0–3 mos	11–13	14–17	18–19		
Infants 4–11 mos	10–11	12–15	16–18	4–<12 mos	12–16
Toddlers 1–2 yrs	9–10	10–13	15–16	1–2 yrs	11–14
Preschoolers 3–5 yrs	8–9	10–13	14	3–5 yrs	10–13
School aged Children 6–13 yrs	7–8	9–11	12	6–12 yrs	9–12
